# Epigenetic Regulation of Mammalian Cardiomyocyte Development

**DOI:** 10.3390/epigenomes8030025

**Published:** 2024-06-29

**Authors:** Isaiah K. Mensah, Humaira Gowher

**Affiliations:** Department of Biochemistry, Purdue University, West Lafayette, IN 47907, USA

**Keywords:** heart, cardiomyocytes, epigenetics, transcription

## Abstract

The heart is the first organ formed during mammalian development and functions to distribute nutrients and oxygen to other parts of the developing embryo. Cardiomyocytes are the major cell types of the heart and provide both structural support and contractile function to the heart. The successful differentiation of cardiomyocytes during early development is under tight regulation by physical and molecular factors. We have reviewed current studies on epigenetic factors critical for cardiomyocyte differentiation, including DNA methylation, histone modifications, chromatin remodelers, and noncoding RNAs. This review also provides comprehensive details on structural and morphological changes associated with the differentiation of fetal and postnatal cardiomyocytes and highlights their differences. A holistic understanding of all aspects of cardiomyocyte development is critical for the successful in vitro differentiation of cardiomyocytes for therapeutic purposes.

## 1. Introduction

The heart is the first organ formed during mammalian development and is critical for the distribution of nutrients and oxygen to other parts of the developing embryo. Following implantation of the embryo, gastrulation occurs to produce the three main germ layers: ectoderm, mesoderm, and endoderm [1,2]. The cells that constitute the heart originate from the mesoderm, with cardiomyocytes being the most abundant cell type and confer contractile activity to the functional heart. The specification of lateral mesoderm cells, a subset of the mesoderm, into the cardiac mesoderm involves several factors, including the effects of signaling pathways, transcription factors, and epigenetic regulation [3,4]. Key transcription factors, such as MESP1, NKX2.5, and GATA4, turn on cardiomyocyte-specific transcriptional programs in the cardiac mesoderm to develop into the first heart field (FHF) and the second heart field (SHF), which are the main reservoirs for cardiomyocytes and form the cardiac crescent, a precursor to the heart [5]. Notably, the pro-epicardial cells, which are also derivatives of the mesoderm, contribute a small percentage of cardiomyocytes to the cardiac crescent [6]. The differentiated mononucleated cardiomyocytes undergo rapid cell division and enlargement to increase cell number and cardiac growth [7,8,9,10]. The transition to postnatal environments triggers the hyperproliferating cardiomyocytes to switch into a hypertrophic state [11], in which mitotic divisions without cytokinesis result in binucleated cardiomyocytes promoting myocardial growth [12,13,14].

In addition to transcriptional programs, several cellular and morphological changes occur in the development of cardiomyocytes. For example, energy utilization switches from glycolysis in early development to oxidative phosphorylation in matured cardiomyocytes. The differentiation of cardiomyocytes from the cardiac mesoderm also involves the establishment of cell–cell coupling, sarcomere assembly, and metabolic shifts that are necessary for the development of fully functioning heart muscles with synchronized contractile activity [15,16,17].

In this review, we describe the cellular and morphological changes that occur in neonatal cardiomyocytes as they mature and transition into postnatal environments. Furthermore, we describe the transcriptional and epigenetic regulation of cardiomyocyte development. We highlight how some of these cardiomyocyte-specific transcription factors interact with epigenetic modulators with a special emphasis on VEZF1, a transcription factor and an insulator binding protein, whose potential role in cardiomyocyte development is understudied. These studies have aided in designing therapeutic strategies against cardiomyocyte deficiency in cardiovascular disease and improved the efficiency of in vitro differentiation methods.

## 2. Cellular Events and Morphological Changes That Regulate the Contractile Activity of Cardiomyocytes

The contractile activity of cardiomyocytes in the fetal and adult heart acts as a central pump for the circulation of oxygen and nutrients in the mammalian embryo [18,19]. Cardiomyocytes are one of the most robust and resilient cell types developed specifically to withstand billions of contractions and relaxation without breaks or self-renewal. The development and maturation of cardiomyocytes can be divided into cellular events, including calcium response, increased ATP consumption, and morphological changes involving the formation and maturation of myofibrils. In prenatal cardiomyocytes, contractile activity is initiated by the oscillations of intracellular calcium levels. The prenatal cardiomyocytes express inositol-3-phosphate receptors (IP3Rs) and ryanodine receptors (RyRs) for the release of calcium from the sarcoplasmic reticulum, as well as the calcium ATPase (SERCA2a) for calcium uptake [20,21]. Contractile activity is achieved by the spontaneous release of IP3Rs and RyRs calcium from the perinuclear sarcoplasmic reticulum [22]. Without active RyRs, IP3Rs cannot produce calcium oscillations independently [23]. The calcium leaks from IP3Rs stimulate neighboring RyRs to release intracellular calcium stores effectively. Thus, in neonatal cardiomyocytes, IP3Rs are the rate-limiting step for calcium release, and therefore, contractile activity depends heavily on the IP3 potential [IP3]i [22,23]. The embryonic cardiomyocytes also possess excitable plasma membrane voltage-activated sodium (L-type) and calcium (T-type) channels, as well as the sodium/calcium (Na^+^/Ca^2+^) exchanger (NCX) [24,25]. The spontaneous release of calcium by the [IP3]i also triggers calcium release by the NCX, generating an inward current and depolarization across the membranes. The depolarization creates an action potential by triggering the voltage-dependent sodium and calcium channels [26]. As cardiomyocytes develop through postnatal phases, calcium release becomes more dependent on the action potential generated for calcium efflux rather than the spontaneous release by the IP3R and RyR channels [26].

The energy utilization of cardiomyocytes also varies significantly between neonatal and postnatal cardiomyocytes. As cardiomyocytes mature, they undergo significant metabolic shits to meet the high energy demands of continuous contraction. Neonatal cardiomyocytes are situated in an anaerobic environment with reduced levels of fatty acids, triggering anaerobic glycolysis as the major source of ATP production for contractile activity [27]. As cardiomyocyte differentiation proceeds, an increase in mitochondrial biogenesis occupies the majority of the cytoplasm in mature cardiomyocytes [28,29]. In postnatal cardiomyocytes, the elevated levels of dietary lipids and the aerobic environment switch ATP production from anaerobic glycolysis to the β-oxidation of fatty acids [30]. The metabolism of lipids generates more ATP to facilitate contractile activity and calcium efflux. A detailed description of the energy unitization differences between fetal and postnatal cardiomyocytes has been previously described [31].

In addition to cellular changes, neonatal cardiomyocytes develop unique morphological structures that aid their function as pumps. These structures, commonly called sarcomeres, comprise the myosin heavy chain, titin, α-actin, and the troponin complex [32]. Of note, the expression of the sarcomere proteins is less in neonatal cardiomyocytes than in postnatal and adult cardiomyocytes. The isoforms of the sarcomere proteins also differ in neonatal and postnatal cardiomyocytes. For example, rat fetal cardiomyocytes express MYH6, which encodes α-MHC. However, adult cardiomyocytes express β-MHC, encoded by MYH7 [33,34,35]. In contrast, humans express β-MHC in both fetal and adult cardiomyocytes [36].

In rats, neonatal cardiomyocytes express longer titin (N2BA), whereas adult cardiomyocytes express a stiff and shorter isoform, N2B [37]. The regulatory and major component of the troponin complex, the troponin I encoded by Tnni3, expresses the slow skeletal muscle isoform of troponin I (ssTnI) in neonatal cardiomyocytes. In contrast, postnatally, the cardiac isoform, cTnI, is expressed [38]. Isoform switches and expression changes of the sarcomere proteins alter the interactions between sarcomeres and other cellular components, including the sarcoplasmic reticulum and mitochondria, to complete the ultrastructure of cardiomyocytes, facilitating the generation of the contractile force needed for blood circulation in the adult organism. A detailed description of fetal to postnatal switches in cardiomyocyte structural proteins has been recently described [15].

## 3. Transcriptional Regulation of Cardiomyocyte Development

**Figure 1:**
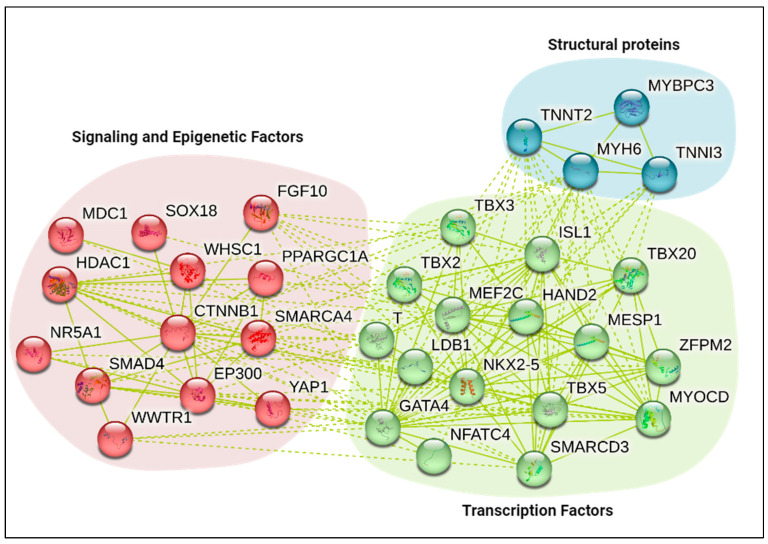
String analysis of proteins involved in cardiomyocyte development. Depiction of the molecular factors that regulate cardiomyocyte development. String analysis was derived from (https://string-db.org/ accessed 4 April 2024). The regulatory factors are grouped as structural proteins (Blue), transcription factors (Green), and signaling and epigenetic factors (Red). The dotted lines represent the link between the separate clusters. Solid lines represent text mining of associated proteins.

Transcription factors are generally characterized by their ability to bind to specific DNA regions and affect transcription. They bind to core promoters, enhancers, or insulators to mediate transcriptional changes. Some major transcription factors involved in cardiomyocyte development include T (Brachyury), Eomes, Mesp1, and Nkx2.5 (Figure 1).

Cardiomyocyte progenitors originate from the mesoderm post-gastrulation. The transcription factor, T (Brachyury), is the master regulator of mesoderm differentiation [39,40,41]. The expression of T is initially present in the presumptive mesoderm and later restricted to the notochord and tail bud [42,43]. The absence of T results in a failure to form mesoderm and causes embryonic lethality [44,45]. Studies in Xenopus reveal that T expression is controlled by FGF signaling [46,47,48].

T and Eomes, a T-box transcription factor expressed in the mesoderm, induce the expression of Mesp1 [49,50,51], a master regulator of cardiac progenitor cells from the mesoderm [52,53,54]. Indeed, increased Mesp1 expression promotes the in vitro differentiation of stem cells into cardiomyocytes from mesoderm cell populations [53,55]. Mesp1 promotes the expression of transcription factors involved in the FHF and SHF development, which include Myocardin, Nkx2.5, Gata4, and Hand2, by directly binding to their promoter regions [53,56,57]. Nkx2.5 and Gata4 are critical for cardiomyocyte differentiation [58,59]. Genetic knockout of Nkx2.5 and Gata4 is embryonic lethal and attributed to cardiac defects [60,61,62]. Nkx2.5 and Gata4 cross-regulate gene expression and interact with each other to regulate the cardiac gene transcription program [58,63,64]. Through its interaction with SMAD4, NKX2.5 is translocated into the nucleus to induce transcription of target genes, coupling BMP signaling with NKX2.5 activity during cardiomyocyte development [65]. Moreover, Gata4 promotes cardiomyocyte proliferation and regeneration in neonatal mice, suggesting a potential role in regeneration following cardiac injury [66].

In the SHF, Mesp1 also induces the expression of Foxh1, a transcription factor necessary for the differentiation of the anterior heart field [67], and is upstream of the transcription factors Isl1 (Islet 1) and Mef2c (Myocyte enhancer factor 2c) [53]. The canonical Wnt signaling pathway also induces the expression of Isl1, emphasizing the role of Wnt signaling in cardiomyocyte development [68,69]. Isl1 is required for the proliferation of Isl1+ cardiomyocyte progenitor cells, and its loss results in severe cardiac development defects accompanied by embryonic lethality [70]. Moreover, Isl1+ cells are multipotent and able to differentiate into all the major cell types of the heart, including cardiomyocytes [71,72]. As a transcription factor, Isl1 regulates the expression of Mef2c, Myocd, and Hand2, which are critical to cardiomyocyte development [73,74]. A detailed overview of Isl1 in cardiomyocyte differentiation has been recently described [75].

The deletion of Mef2c is also embryonic lethal, with severe cardiovascular defects with no impact on endothelial cell differentiation [76,77]. The expression of Mef2c marks the commitment of cells to the cardiac lineage during embryogenesis [78]. Mef2c binds to and turns on gene expression in cardiomyocyte structure and function, including α-MHC, Tnnt2, desmin, muscle creatine kinase, etc. [79,80]. MEF2C can physically interact with GATA4 and function as a coactivator to promote the expression of cardiomyocyte-specific genes [81,82]. In addition to the core transcription factors, other factors, such as those belonging to the T-box family (Tbx5, Tbx2, Tbx3, Tbx20), Hand1/Hand2, and Myocd, all play essential roles in the maturation and contractile activity of cardiomyocytes [83,84].

## 4. Insulator Activity of Transcription Factors

**Figure 2:**
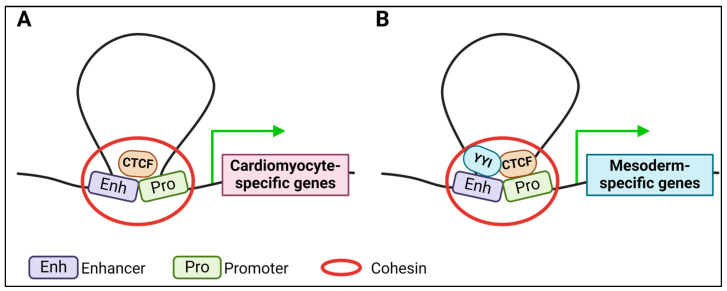
Roles of insulator binding proteins in cardiomyocyte development. (**A**) Depiction of CTCF binding to insulators to mediate enhancer–promoter interactions facilitated by cohesion, to enhance expression of cardiomyocyte-specific genes. (**B**) CTCF also recruits Ying yang 1 (YY1) to mediate expression of mesoderm-specific genes. Grean arrow: Active gene expression. Created in BioRender.com.

While most transcription factors (TFs) affect gene expression by binding to the promoter or enhancer region of genes, some TFs function by binding to insulator elements on the DNA. Enhancers are cis-regulatory elements that amplify transcriptional initiation at gene promoters. Insulators were generally defined as cis-regulatory elements between enhancers and promoters, where insulator binding factors bind to block enhancer–promoter interactions and impede transcription [85]. However, several other functions of insulators were discovered, including spreading histone modifications, promoting long-distance contact and gene regulation, and influencing chromatin structure [86]. One of the well-studied insulator-binding transcription factors is CTCF (CCCTC-binding factor) [87]. CTCF plays diverse roles in regulating gene expression, including functioning as a barrier to prevent the spread of heterochromatin, establishing boundaries between topologically associated domains (TADs), occluding enhancer–promoter interactions, and promoting enhancer–promoter interactions [88,89]. CTCF plays a critical role in embryonic development, its loss causing embryonic lethality due to a wide range of developmental abnormalities [90,91]. Studies using conditional deletion of CTCF during development revealed that CTCF facilitates local chromatin interactions to promote gene expression in cardiac development [92] (Figure 2A). CTCF was also shown to maintain the global chromatin loop architecture of cardiomyocytes in response to stress [93]. Another common insulator protein, Ying yang 1 (YY1), which also interacts with CTCF, is required to commit mesoderm cells into cardiomyocytes and promote overall cardiac development [94,95,96,97,98] (Figure 2B). Despite these established roles in cardiomyocyte development, more studies are needed on the mechanism and function of insulator-binding transcription factors in cardiomyocyte development.

## 5. The Emerging Role of VEZF1 in Cardiac Development

**Figure 3:**
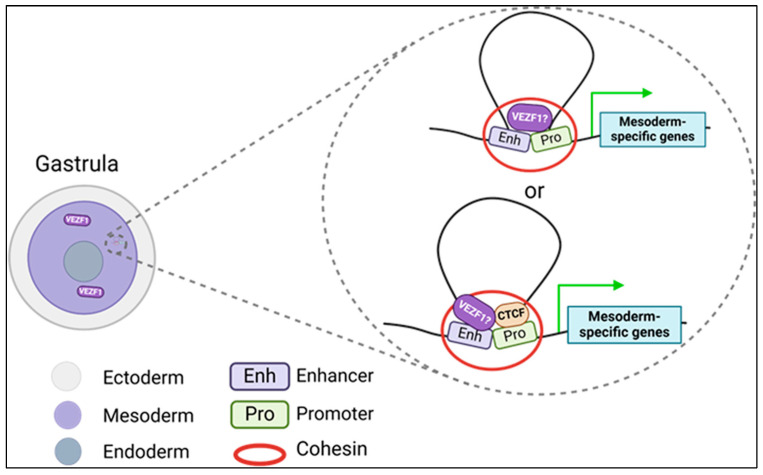
Potential function of VEZF1 in the mesoderm. VEZF1 is predominantly expressed in the mesoderm and potentially retains insulator binding activity during gastrulation to enhance mesoderm-specific gene expression. This mechanism of promoting gene expression could be independent or in concert with other insulator binding proteins, including CTCF. Grean arrow: Active gene expression. Created in BioRender.com.

Vascular endothelial zinc finger 1 (Vezf1) is a transcription factor with newly identified roles in cardiac development. VEZF1 contains six C2H2 motifs, belongs to the Krüppel zinc finger family, and is highly conserved among vertebrates. Multiple roles for VEZF1 in the regulation of gene expression are known. For example, VEZF1 contains a proline-rich transactivation domain that promotes gene expression [99,100], VEZF1 modulates the elongation function of RNA polymerase affecting both transcription and splicing [101], and VEZF1 protects its binding sites against DNA methylation, which potentially allows transcription factor binding to promote gene expression [102]. The loss of Vezf1 in stem cells reduces global DNA methylation levels via the decreased expression of the catalytically active spliced isoform of Dnmt3b [103], which can cause adverse contingent changes in gene expression. VEZF1 functions as an insulator binding protein to protect sites against DNA methylation and prevents the spread of heterochromatin [102,104,105]. During mouse development, Vezf1 is detected in the extraembryonic and embryo mesoderm and later in mesodermal lineages [106]. However, the precise role of Vezf1 in mesoderm and mesodermal lineage differentiation is still unclear. It is possible that VEZF1 functions at insulators to promote mesoderm gene expression (Figure 3). The homozygous knockout of Vezf1 is embryonic lethal during mouse development [107]. While most studies have focused on the role of VEZF1 in forming endothelial and hematoendothelial lineages [108,109,110,111], Vezf1 is also important for cardiomyocyte development and function. For instance, the loss of Vezf1 in Zebrafish significantly decreases cardiomyocytes and impairs cardiac contractile function [112]. Additionally, mutations in Vezf1 were found to be highly associated with dilated cardiomyopathy [113], further establishing the role of VEZF1 in cardiomyocyte development and function.

## 6. Epigenetic Regulation of Cardiomyocyte Differentiation

Epigenetic mechanisms include the role of DNA methylation, histone tail modifications, chromatin structure, and noncoding RNAs in the regulation of gene expression without changing the cognate DNA sequences. Epigenetic regulation is a key driver of developmental programs and cellular differentiation [114]. Changes in DNA methylation and histone tail modifications at the cis-regulatory elements of a gene (promoters and enhancers) impact its expression [115]. Similarly, an organized chromatin structure mediates the assembly of transcriptionally active and inactive regions, creating high-density hubs with enhanced ability to share the transcriptional machinery. Noncoding RNA can act as scaffolds to stabilize large protein complexes containing histone and DNA-modifying enzymes and transcription factors. Based on the protein complexes they recruit, noncoding RNAs mediate gene activation or repression. Moreover, noncoding RNA expressed within a gene can impact cognate gene expression in cis or migrate to distantly placed genes and act in trans. Several epigenetic mechanisms regulate the transcription factors and signaling molecules involved in cardiovascular development.

## 7. DNA Methylation

**Figure 4:**
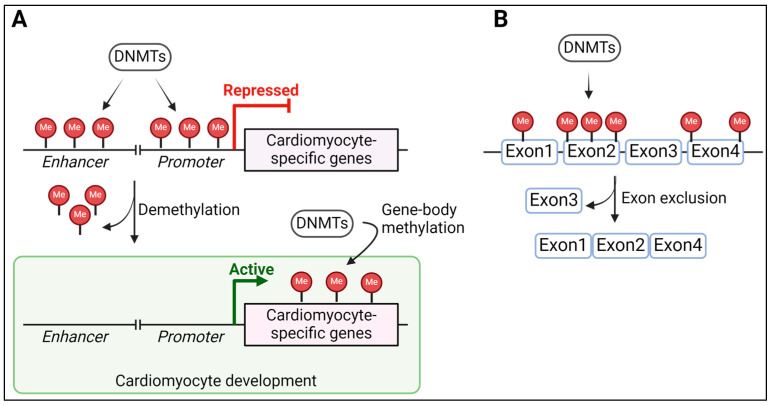
Roles of DNA methylation in cardiomyocyte development. (**A**) Illustration of DNA methylation in enhancers and promoters of cardiomyocyte genes that are erased during cardiomyocyte development. (**B**) Schematic of gene body DNA methylation on alternative splicing of cardiomyocyte genes. DNMTs: DNA methyltransferases, Me: Methyl groups. Created in BioRender.com.

DNA methylation and methyltransferases play crucial roles in development and disease [116,117]. Misregulation of DNA methylation is implicated in cardiac diseases [118,119]. In mammals, DNA methylation is the deposition of a methyl group by DNMTs (DNA methyltransferases) at the 5′ position of cytosine, mainly in a CpG sequence context [120]. There are four primary DNA methyltransferases, DNMT1, DNMT3A, DNMT3B, and DNMT3L, responsible for DNA methylation in higher eukaryotes. While DNMT3A and DNMT3B establish new DNA methylation patterns in the genome, DNMT1 copies DNA methylation from the parent to the daughter strand at CpG sites following DNA replication. DNMT3L is catalytically inactive and functions as an accessory protein to facilitate the catalytic activity of DNMT3A and DNMT3B. DNA methylation can promote or repress gene expression depending on its positioning on a gene. DNA methylation at CpG-poor promoter and enhancer regions of differentially methylated developmental genes is often associated with the repression of gene expression [121,122]. Genome-wide DNA methylation profiling of developing mouse embryos revealed that about 79 genes involved in cardiomyocyte development, including Myocd, Mef2c, Hand1, and Tbx20, are differentially methylated. Notably, the methylation status of these genes correlated with their expression, where their increased expression corresponds to decreased methylation at gene enhancers and promoters, emphasizing the role of DNA methylation in cardiomyocyte development [123]. In a similar study using human ESC-derived cardiomyocytes, increased expression of cardiomyocyte-specific genes (mostly involved in cardiac muscle contraction and sarcomere-related transcripts) correlated with reduced promoter DNA methylation [124]. Another study highlighted the importance of DNMT1 by showing global demethylation in DNMT1 knockdown cells, including at the promoters of Myh6, Tnnc1, Tnni3, Tnnt2, Nppa, and Nppb, that corresponds with their increased expression [125] (Figure 4A). The knockdown of DNMT3A reduces the expression of cardiomyocyte contractile proteins, including Myh7, and causes defects in cardiomyocyte metabolism, revealing a role of DNA methylation and DNMTs in maintaining cardiomyocyte homeostasis [126]. However, future research is needed to elucidate regulatory mechanisms, identifying novel target genes during cardiomyocyte differentiation, and uncovering the functional consequences of methylation changes during development.

The role of DNA methylation in alternative splicing has been thoroughly investigated. Alternative splicing is a major contributor to the complexity and diversity of the proteome, generating distinct functional proteins from the same gene. The modes by which alternative splicing diversifies mRNA include exon skipping, where specific exons are removed from the mRNA, utilization of alternative splice sites, intron retention, and a choice between unrelated exons [127]. Alternative splicing can occur co-transcriptionally and, hence, is regulated by factors such as DNA methylation that slow down the rate of RNA Pol II during transcription [128]. For instance, DNA methylation within gene bodies contributes positively to alternative splicing [129] (Figure 4B). During cardiomyocyte differentiation, alternatively spliced genes were shown to be associated with differentially methylated regions [130]. Demethylated regions localized within gene bodies of cardiomyocyte genes, including cardiomyocyte structural proteins Myh6 and Myh7, are critical to the perinatal isoform switch required for cardiomyocyte maturation [131]. The role of DNMT1 in the isoform switch of cardiac-specific genes, including Gata4, Myh7, Tmp1 and Tpm2, is underscored by aberrant exon splicing in the absence of DNMT1 [125].

## 8. Histone Modifications

The mammalian genome is packaged into nucleosomes consisting of DNA wrapped around octamers of two copies each of four core histones to form nucleosomes [132]. The core histones have unstructured N’ terminal tails liable to post-translational modifications, with the highly conserved histone H3 and H4 tails constituting the most studied post-translational histone modifications [133,134]. The enzymes that deposit the post-translational modification on histone tails are often called writers, those that remove the marks are called erasers, and those that bind to these modifications as readers [135]. Typically, the acetylation of histone tails is associated with cognate gene activation, whereas deacetylation is associated with cognate gene repression [136,137]. However, histone tail methylation is context-dependent and can promote or inhibit gene expression. For instance, the methylation of H3K9 and H3K27 histone tail residues is associated with gene repression, whereas methylation of H3K4 and H3K36 is associated with gene activation [138].

**Figure 5:**
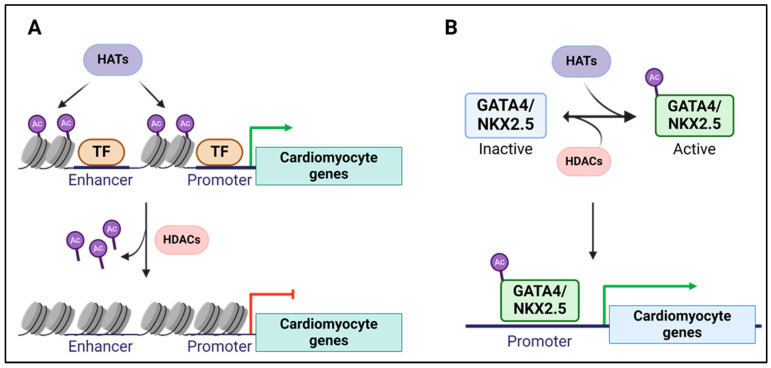
Roles of histone acetyl modifying enzymes in cardiomyocyte development. (**A**) Schematic of histone acetylation by HATs in enhancers and promoters of cardiomyocyte genes that mediate activation of gene expression. HDACs erase histone tail acetylation. (**B**) Illustration of cardiomyocyte-specific transcription factor activation by HATs to enhance their binding to DNA to promote cardiomyocyte gene expression. HATS: Histone acetyltransferase; HDACs: Histone deacetylases; Ac: Acetyl group; TF: transcription factor; Green arrow: Activation of gene expression; Red arrow: repression of gene expression. Created in BioRender.com.

Histone modifications are crucial in cardiomyocyte development [139,140]. For instance, p300, a histone acetyltransferase, is required for cardiac development and enhances the expression of αMHC [141,142,143]. p300 acetylates histones around Gata4, Nkx2.5, and Mef2c, critical transcription factors for cardiomyocyte development, to promote their expression [144] (Figure 5A). Beyond histone tail acetylation, p300 physically interacts with and acetylates Nkx2.5 and Gata4, enhancing their DNA binding activity to promote the expression of cardiomyocyte-specific genes [145,146] (Figure 5B). Histone deacetylases, HDAC1 and HDAC2, are also crucial in cardiac development [147]. The overexpression of HDACs inhibits cardiomyocyte development, whereas their inhibition with trichostatin A promotes the differentiation of mesodermal cells into cardiomyocytes [148]. Moreover, HDACs repress Mef2, and the repression can be relieved by proteolytic degradation of HDAC2 [149,150]. HDAC2 interacts with HOPX (homeodomain factor), which interacts with and deacetylates Gata4. The deacetylation of Gata4 reverts the upregulation of Gata4 target genes and cardiomyocyte proliferation [151].

The methylation of histones also plays an essential role in cardiomyocyte development. Mutations in genes that methylate H3K4 and H3K27 cause congenital heart disease [152,153]. The loss of the histone methyltransferase, WHSC1, is embryonic lethal, with defects in cardiovascular development during embryogenesis. Moreover, WHSC1 interacts with the cardiac-specific transcription factor Nkx2.5 and is implicated in the repression of Nkx2.5 target genes [154]. Ezh2, a member of the PRC2 histone methyltransferase complex, stabilizes the expression of cardiac genes and prevents cardiac pathology [155]. The histone demethylase UTX (Ubiquitously expressed tetratricopeptide repeat gene on the X chromosome) demethylates trimethylated H3K27 to promote the expression of cardiac genes [153]. UTX also interacts with Nkx2.5, Gata4, and Tbx5 to promote cardiac-specific gene expression [153].

Histone ubiquitination is a post-translational modification where ubiquitin, a small regulatory protein, is added to lysin residues of histone [156]. The monoubiquitination of histone H2A is associated with transcriptional repression, mediated through the polycomb repressive complex 1 (PRC1), which plays a crucial role in maintaining the silenced state of developmental genes during cardiomyocyte development [157,158]. The monoubiquitination of histone H2B is associated with transcriptional activation. H2B ubiquitination is catalyzed by the RNF20/RNF40 complex and is important for RNA polymerase II elongation and the subsequent methylation of histone H3 at lysine 4 (H3K4me3), a mark of active transcription [159]. For example, the monoubiquitination of H2B (H2Bub1) was shown to regulate the expression of cilia genes during *Xenopus* and mice heart development and is necessary for gene expression and maintenance of adult cardiomyocytes [160,161]. Additionally, human genetic studies have shown that mutations in RNF20/40 contribute to congenital heart disease, a structural abnormality present at birth [157]. However, the mechanism by which histone ubiquitination at cardiomyocyte-specific genes affects cardiac development is largely unknown.

SUMOylation of histones and transcription factors involves the attachment of a small ubiquitin-like modifier (SUMO) protein, which influences chromatin dynamics gene expression and regulates the activity of transcription factors [162,163]. SUMOylation of NKX2.5 and GATA4 strongly enhances their nuclear localization, DNA binding, and transcriptional activity at cardiomyocyte-specific genes [164,165].

## 9. Noncoding RNAs in Cardiomyocyte Development

Noncoding RNAs (ncRNA) also play crucial roles in regulating the expression of genes necessary for cardiomyocyte development. In general, noncoding RNAs are characterized as functional RNAs that do not encode proteins [166]. There are several classes of noncoding RNAs grouped into the infrastructural ncRNAs, such as ribosomal and transfer RNAs, and the regulatory ncRNAs, including micro, small interfering (siRNAs), Piwi-interacting RNAs (piRNAs), and long noncoding RNAs (lncRNA) [167]. Of these, RNAs (lncRNA) and microRNAs (miRNA) are mostly studied in cardiomyocyte development [168,169,170].

MicroRNAs (miRNAs) are typically short ~21–22 nucleotide RNA molecules that inhibit translation or promote mRNA degradation by binding to the 3′ untranslated region of the target mRNA [171,172]. Several miRNAs are implicated in almost all stages of cardiomyocyte generation during early development, including mesoderm differentiation, development of cardiac mesoderm, cardiomyocyte proliferation, and cardiomyocyte maturation. miR-1 is the most abundant miRNA expressed in mammalian hearts [173], with significant roles in cardiomyocyte development and maturation. The overexpression of miR-1 reduces cardiac growth and cardiomyocyte proliferation, potentially via inhibiting the expression of cardiac-specific transcription factors like Hand2 and Nkx2.5 [174,175]. Through its effects on the WNT and NOTCH signaling pathways, miR-335 was shown to positively regulate the formation of cardiac mesoderm, where overexpression of miR-335 led to an increase in the expression of Brachyury, Gata4, and Nkx2.5. Interestingly, the loss of miR-335 showed opposite effects on the expression of cardiac mesoderm genes and overall cardiomyocyte differentiation [176]. Additionally, miR-200c has been shown to repress differentiation of human ESCs (hESCs) into cardiomyocytes, potentially through its inhibitory effect on key genes, such as GATA4, SRF, and TBX5, necessary for cardiomyocyte differentiation [177]. A comprehensive list of miRNAs in cardiomyocyte development has been previously described [169,178].

Unlike miRNAs, lncRNAs are longer and comprise at least 200 nucleotides in the RNA transcript [179]. lncRNAs can be further divided into antisense, sense-intronic, intergenic, and bidirectional lncRNA classes, all of which are crucial for cardiomyocyte development. Typically, lncRNAs function at all levels of gene regulation, including the modulation of chromatin structure and function, the transcription of genes, RNA splicing, and translation [180]. The diversity in function of lncRNAs stems from their ability to interact with DNA, RNA, and proteins. lncRNAs function in all aspects of development, with established roles in cardiomyocyte and heart development. For instance, two lncRNAs flank around the Hand2 gene, one upstream of Hand2 (*Upperhand*) and the other several kilobases downstream of Hand2 (*Handsdown*). The deletion of the *Upperhand* is embryonic lethal with heart defects, emphasizing its role in cardiac development [181,182]. Mechanistically, *Upperhand* was shown to regulate the expression of Hand2 positively by maintaining an active promoter of Hand2 and facilitating transcription by RNA Pol II [181,182]. Conversely, *Handsdown* negatively regulates the expression of Hand2 by looping Hand2 promoters with upstream regulators mediated by CTCF, thereby occluding the promoter from active gene transcription by RNA pol II [183]. Several other lncRNAs, such as CARMA and Platr4, also regulate cardiomyocyte differentiation by modulating the Notch and Hippo signaling pathways, respectively [184,185]. More recently, another lncRNA, Charme, emerged as significant for cardiomyocyte proliferation, via the formation of active nuclear condensates containing RNAs critical for cardiomyocyte development [186].

## 10. ATP-Dependent Chromatin Remodelers

ATP-dependent chromatin remodelers use energy from ATP hydrolysis to alter nucleosome packaging by displacing or exchanging histones to elicit gene activation or repression [187]. The chromatin remodeling complex, SWI/SNF, is well-studied in cardiac development [188]. Notably, BRG1/BAF (Brahma-related gene 1/Brahma-associated factor) interacts with cardiomyocyte-specific transcription factors, including Gata4, Nkx2.5, and Tbx5 via Baf60c (BAF subunit enriched in cardiac cells) to promote the induction of mesoderm cells into beating cardiomyocytes [189]. Baf60c also interacts with MYOCD to coregulate MYOCD target genes that promote cardiomyocyte development [190]. Indeed, BRG1 is required to develop mouse and zebrafish hearts and genetically interacts with Nkx2.5 and Tbx5 to regulate cardiac-specific gene programs [191]. Additionally, BRG1 regulates cardiomyocyte growth and differentiation by maintaining Bmp10 expression, activating β-MHC expression in neonatal hearts, and suppressing α-MHC expression in adult cardiomyocytes [192].

## 11. Conclusions and Outcomes

Despite extensive research in cardiomyocyte development, significant gaps remain. The epigenome during cardiomyocyte development is understudied, partly due to the emphasis on identifying key cardiac transcription factors and signaling molecules. Cell differentiation into cardiomyocytes is dynamic and requires the commitment of specific mesoderm populations toward cardiomyocytes. Although many reports describe the role of specific epigenetic modifications in the transcriptional regulation of some cardiac-specific genes, comprehensive studies profiling the entire epigenome landscape, including histone modifications, chromatin remodeling, and noncoding RNAs, are required to fully understand the dynamic regulation of the epigenome in the process. Using multi-omic approaches and in vitro differentiation models from stem cells will be key in uncovering the dynamic epigenetic changes during cardiomyocyte development. Additionally, knowledge of the epigenome in cardiomyocyte development will provide diverse alternative modules for therapeutic strategies toward cardiomyocyte regeneration. We have provided a comprehensive overview of key processes involved in cardiomyocyte development. We highlight the dynamic interplay of cellular events, morphological changes, transcriptional and epigenetic regulation during cardiomyocyte development. Understanding these key processes will ultimately reveal the molecular switches that regulate cardiomyocyte proliferation and growth, knowledge of which is urgently needed to curb the high mortality rates associated with cardiomyocyte insufficiency in cardiac diseases.
